# Awareness of and Beliefs About Naloxone Among Adults

**DOI:** 10.1001/jamahealthforum.2025.1867

**Published:** 2025-06-27

**Authors:** S. Michaela Rikard, Kunal Doshi, Gery P. Guy, Kristine M. Schmit

**Affiliations:** 1 Division of Overdose Prevention, Centers for Disease Control and Prevention, Atlanta, Georgia; 2 Public Health and General Preventive Medicine Residency, Emory University, Atlanta, Georgia; 3 Addiction Medicine Fellowship, Loma Linda University, Loma Linda, California

## Abstract

This cross-sectional study describes the level of naloxone information and availability and general attitudes about illicit drug use across different demographic groups in the US.

## Introduction

Naloxone and other opioid overdose reversal medications can save lives when given in time.^1^ Despite increases in pharmacy-dispensed naloxone,^2^ additional naloxone distribution is needed through both community-based and pharmacy sources.^3^ Reported barriers include cost, stigma, and lack of knowledge and training related to naloxone access and use.^4^ The objective of this study was to analyze US adults’ awareness of and beliefs about naloxone and its availability.

## Methods

Data were obtained from round 2 (collected October-November 2023) of the National Center for Health Statistics Rapid Surveys System. Estimates were weighted to be nationally representative.^5^ This activity was reviewed by the Centers for Disease Control and Prevention (CDC), deemed research not involving human participants, and conducted consistent with applicable federal law and CDC policy.

Whether adults had heard of naloxone, whether they currently carried naloxone, and their reasons for not carrying naloxone were analyzed overall and by demographic characteristics. Estimates of whether adults currently carried naloxone, were aware of naloxone’s availability, and agreed with naloxone availability in various locations were analyzed overall and stratified by their agreement or disagreement that persons are to blame for their drug use.

SAS, version 9.4 (SAS Institute), was used for analyses, and survey weights were applied. Two-sided *P* < .05 was considered statistically significant.

## Results

A total of 7046 adults (3796 female [weighted 51.2%]) responded to the 2023 survey. Overall, 71.0% of adults reported having heard of naloxone, of whom 5.6% currently carried naloxone (Table 1). Older adults, females, those with higher educational level, those residing in nonmetropolitan areas, and those with higher household incomes had a higher prevalence of having heard of naloxone, whereas adults of Hispanic, non-Hispanic Black, and other race and ethnicity and those residing in the South and West had a lower prevalence. Adults 65 years or older, those with a bachelor’s degree, and those with higher household incomes reported a lower prevalence of carrying naloxone, while Hispanic adults reported a higher prevalence. Reasons reported for not carrying naloxone included not thinking it works (9.2%), not knowing how to use it (60.1%), and not knowing where to get it (67.3%); significant differences by demographic characteristics were found.

**Table 1.** Awareness of Naloxone and Factors Associated With Carrying Naloxone by Demographic Characteristics^a^

Characteristic	Adults, weighted % (95% CI)				
	Heard of naloxone/Narcan (unweighted n = 7016)	Currently carry naloxone/Narcan (unweighted n = 5258)^b^	Why do you think people do not carry naloxone/Narcan?^c^		
			Do not think it works (unweighted n = 4778)^c^	Do not know how to use it (unweighted n = 4821)^c^	Do not know where to get it (unweighted n = 4834)^c^
Overall	71.0 (69.6-72.4)	5.6 (4.8-6.4)	9.2 (8.2-10.2)	60.1 (58.4-61.8)	67.3 (65.6-68.9)
Age group, y					
18-34 [Reference]	63.2 (59.9-66.4)	7.4 (5.6-9.7)	10.8 (8.5-13.6)	68.2 (64.3-72.0)	74.5 (70.8-78.0)
35-49	70.6 (67.8-73.3)^d^	7.1 (5.3-9.3)	7.5 (5.8-9.5)^d^	59.8 (56.3-63.3)^d^	68.7 (65.2-72.0)^d^
50-64	74.2 (71.7-76.5)^d^	5.6 (4.2-7.3)	8.5 (6.7-10.5)	55.2 (51.9-58.5)^d^	64.0 (60.8-67.1)^d^
≥65	78.3 (76.0-80.5)^d^	2.1 (1.3-3.1)^d^	9.8 (8.1-11.8)	57.0 (54.0-59.9)^d^	61.8 (58.8-64.6)^d^
Sex					
Male [reference]	68.9 (66.8-71.0)	6.0 (4.8-7.4)	8.5 (7.1-10.0)	56.9 (54.4-59.4)	64.0 (61.5-66.4)
Female	73.1 (71.2-74.9)^d^	5.2 (4.2-6.3)	9.8 (8.5-11.3)	63.0 (60.7-65.3)^d^	70.2 (68.0-72.4)^d^
Race and ethnicity^e^					
Black, non-Hispanic	62.8 (58.1-67.4)^d^	6.2 (3.9-9.3)	14.2 (10.2-19.0)^d^	58.7 (53.0-64.2)	65.6 (59.8-71.1)
Hispanic	49.9 (46.0-53.9)^d^	9.3 (5.9-13.9)^d^	13.9 (10.1-18.3)^d^	70.9 (65.4-76.0)^d^	79.9 (75.2-84.1)^d^
White, non-Hispanic [reference]	80.3 (78.8-81.7)	4.8 (4.0-5.7)	7.9 (6.9-9.1)	58.2 (56.2-60.1)	64.6 (62.6-66.5)
Other, non-Hispanic^f^	58.7 (53.0-64.2)^d^	6.4 (3.4-11.0)	5.7 (3.1-9.4)	63.9 (56.9-70.4)	75.7 (69.5-81.2)^d^
Educational level					
≤High school diploma [reference]	61.8 (59.1-64.4)	6.8 (5.2-8.7)	11.4 (9.5-13.6)	58.1 (54.6-61.6)	64.9 (61.6-68.2)
Some college	75.7 (73.3-77.9)^d^	5.6 (4.2-7.3)	9.8 (8.1-11.9)	59.9 (56.9-62.9)	67.3 (64.3-70.1)
≥Bachelor’s degree	77.5 (75.5-79.4)^d^	4.4 (3.4-5.6)^d^	6.8 (5.6-8.2)^d^	62.0 (59.5-64.5)	69.3 (66.8-71.7)^d^
Urbanicity					
Metropolitan [reference]	70.2 (68.7-71.7)	5.6 (4.7-6.6)	9.3 (8.3-10.5)	60.9 (59.1-62.8)	68.0 (66.2-69.7)
Nonmetropolitan	76.2 (72.6-79.5)^d^	5.4 (3.7-7.6)	8.3 (5.9-11.3)	55.3 (50.9-59.7)^d^	63.4 (59.0-67.6)^d^
Region					
Northeast [reference]	75.8 (72.5-78.9)	6.4 (4.6-8.6)	8.6 (6.7-10.8)	61.7 (57.5-65.8)	65.8 (61.9-69.6)
Midwest	78.8 (76.2-81.2)	4.8 (3.5-6.5)	8.1 (6.3-10.2)	59.4 (55.9-62.8)	63.7 (60.4-67.0)
South	66.2 (63.7-68.6)^d^	4.8 (3.6-6.3)	10.0 (8.3-12.0)	58.9 (56.1-61.6)	68.8 (65.9-71.5)
West	68.5 (65.6-71.3)^d^	6.7 (4.8-9.0)	9.4 (7.4-11.8)	61.5 (58.0-64.9)	69.8 (66.3-73.2)
Household income					
<200% FPL [reference]	60.5 (57.7-63.4)	7.7 (5.9-9.7)	12.3 (10.0-14.9)	60.3 (56.6-63.8)	68.9 (65.4-72.1)
≥200 to <400% FPL	72.1 (69.3-74.7)^d^	5.3 (3.8-7.2)	10.4 (8.6-12.4)	61.0 (57.7-64.3)	69.4 (66.1-72.5)
≥400% FPL	77.8 (75.9-79.6)^d^	4.5 (3.5-5.7)^d^	6.9 (5.7-8.3)^d^	59.5 (57.1-61.9)	65.3 (62.8-67.7)^d^

Abbreviation: FPL, federal poverty level.

^a^ Data from the National Center for Health Statistics Rapid Surveys System.^5^ Missing responses, including those coded as “refused” or “don’t know,” were excluded from the respective analyses. Additional information on the data collection and survey weighting is available in the survey description.

^b^ This question was asked only among respondents who answered yes to the question, “Have you ever heard of the medication naloxone, also known as Narcan, which can be used to reverse an opioid overdose?”

^c^ These questions were asked only among respondents who answered no to the question, “Do you currently carry naloxone or Narcan?”

^d^ Difference from the reference group was significant based on 2-sided *P* < .05. Statistical significance was determined based on coefficients from an unadjusted logistic regression model of each demographic variable.

^e^ Self-reported by participants. Missing data for race and ethnicity were imputed.

^f^ Includes American Indian or Alaska Native, Asian, Native Hawaiian or Other Pacific Islander, and multiple races.

Among adults who agreed that persons were to blame for their drug use, 4.3% currently carried naloxone compared with 8.3% among those who disagreed (Table 2). Awareness of where naloxone is available was highest for pharmacies (63.8%) and lowest for community harm-reduction organizations (31.7%). Agreement on where naloxone should be available was highest for college or university campuses (83.7%) and lowest for places of worship (54.0%). Both awareness of naloxone availability and agreement on where naloxone should be available were significantly lower among those who agreed persons were to blame for their drug use.

**Table 2.** Awareness of Naloxone Availability and Attitudes About Expanding Access Stratified by Agreement That Persons Are to Blame for Their Drug Use^a^

	Adults, weighted % (95% CI)			*P* value for difference between agree and disagree categories^d^
	Overall (unweighted N = 5258)	Among adults who agree that persons are to blame for their drug use (unweighted n = 3409)^b^	Among adults who disagree that persons are to blame for their drug use (unweighted n = 1781)^c^	
Currently carry naloxone/Narcan^e^	5.6 (4.8-6.4)	4.3 (3.4-5.3)	8.3 (6.7-10.0)	<.001
Are aware that naloxone is available at the following locations^e^				
Pharmacies	63.8 (62.2-65.4)	62.0 (60.0-64.0)	68.0 (65.3-70.7)	<.001
Over the counter without prescription	52.4 (50.7-54.0)	49.7 (47.7-51.7)	58.3 (55.4-61.1)	<.001
Physician offices	47.0 (45.3-48.6)	45.8 (43.7-47.9)	49.3 (46.5-52.1)	.05
Health departments	43.0 (41.2-44.7)	40.6 (38.6-42.7)	48.2 (45.2-51.2)	<.001
Community harm-reduction organizations	31.7 (30.1-33.2)	26.3 (24.5-28.1)	42.6 (39.8-45.5)	<.001
Agree that naloxone/Narcan should be available at the following locations^e^				
College or university campuses	83.7 (82.4-84.9)	78.9 (77.1-80.5)	92.9 (91.1-94.3)	<.001
High schools	78.1 (76.7-79.5)	72.8 (70.9-74.7)	88.0 (86.0-89.9)	<.001
Public libraries	59.1 (57.5-60.8)	51.4 (49.3-53.4)	74.0 (71.4-76.5)	<.001
Elementary and middle schools	56.5 (54.8-58.1)	51.2 (49.2-53.3)	66.3 (63.4-69.1)	<.001
Businesses	55.5 (53.8-57.1)	49.1 (47.1-51.1)	67.4 (64.7-70.0)	<.001
Places of worship	54.0 (52.4-55.7)	46.0 (43.9-48.1)	69.1 (66.4-71.7)	<.001

^a^ Data from the National Center for Health Statistics Rapid Surveys System.^5^ Missing responses including those coded as “refused” or “don’t know” were excluded from the respective analyses. Additional information on the data collection and survey weighting is available in the survey description.

^b^ Based on a response of “strongly agree” or “somewhat agree” to the question, “How much do you agree or disagree with the following statement? I think that a person who misuses prescription opioids or uses illegal drugs, such as cocaine or heroin, is to blame for his or her drug use.”

^c^ Based on a response of “strongly disagree” or “somewhat disagree” to the question, “How much do you agree or disagree with the following statement? I think that a person who misuses prescription opioids or uses illegal drugs, such as cocaine or heroin, is to blame for his or her drug use.”

^d^ Two-sided *P* values were calculated using Rao-Scott χ^2^.

^e^ These questions were asked only among respondents who answered yes to the question, “Have you ever heard of the medication naloxone, also known as Narcan, which can be used to reverse an opioid overdose?”

## Discussion

This study found that, although most adults reported having heard of naloxone, few carried naloxone. Those reporting highest awareness of naloxone also reported lowest prevalence of carrying naloxone, potentially indicating that different factors affect these outcomes. Younger adults, females, Hispanic adults, and those residing in metropolitan areas had a higher prevalence of not knowing how to use or where to get naloxone; non-Hispanic Black and Hispanic adults had higher prevalence of thinking naloxone does not work. Blaming persons for their drug use was associated with lower prevalence of carrying naloxone and having awareness of and agreement with naloxone availability in various locations. Overall, naloxone awareness and agreement with additional access were high, but barriers were reported, such as not knowing how to use or where to get naloxone. Public health initiatives that increase naloxone access and training could help save lives from overdose.

Study limitations include generalizability only to noninstitutionalized civilian adults, potential recall bias, and lower response rates, which may contribute to nonresponse bias. Despite these limitations, this study identified opportunities to expand naloxone access and education and highlighted the importance of addressing stigma surrounding persons who use drugs.

## Notes

Supplement.
Data Sharing Statement
