# Supplementary material for: Awareness of and Beliefs About Naloxone Among Adults
Source: JAMA Health Forum. 2025 Jun 27;6(6):e251867. doi: 10.1001/jamahealthforum.2025.1867 (PMC12205397; doi:10.1001/jamahealthforum.2025.1867)
Supplement: Supplement. — Data Sharing Statement [file jamahealthforum-e251867-s001.pdf]

Data Sharing Statement

Rikard. Awareness of and Beliefs About Naloxone Among Adults. *JAMA Health Forum*. Published June 27, 2025. doi:10.1001/jamahealthforum.2025.1867

Data
Data available: Yes
Data types: Data (not involving human participants)
How to access data: Data are available publicly online at: https://www.cdc.gov/nchs/rapid-surveys/documentation/round-2.html
When available: With publication

Supporting Documents
Document types: None

Additional Information
Who can access the data: Data are available publicly
Types of analyses: For any purpose
Mechanisms of data availability: Available online
